# Detection of *Wuchereria bancrofti* in the city of São Luís, state of Maranhão, Brazil: New incursion or persisting problem?

**DOI:** 10.1371/journal.pntd.0011091

**Published:** 2023-01-30

**Authors:** Tatiane Alexandre de Araújo, Alessandra Lima de Albuquerque, Danielle Cristina Tenório Varjal de Melo, Eloína Maria de Mendonça Santos, André Luiz Sá de Oliveira, Constância Flávia Junqueira Ayres, Cláudia Maria Fontes de Oliveira

**Affiliations:** 1 Instituto Aggeu Magalhães—Fundação Oswaldo Cruz, Departamento de Entomologia, Cidade Universitária, Recife, Pernambuco, Brasil; 2 Instituto Aggeu Magalhães—Fundação Oswaldo Cruz, Núcleo de Estatística e Geoprocessamento, Cidade Universitária, Recife, Pernambuco, Brasil; University of Liverpool, UNITED KINGDOM

## Abstract

**Background:**

The elimination of lymphatic filariasis (LF) from Brazil by 2020 was not accomplished; however, this goal can be achieved in the upcoming years with the assistance of specific strategies. The surveillance of LF can be performed using molecular xenomonitoring (MX), a noninvasive method used to infer the presence of the parasite in the human population. Herein, São Luís (state of Maranhão) was the first city to be investigated to identify whether LF transmission in Brazil has been interrupted and if there were any new incursions.

**Methodology/Principal findings:**

Mosquitoes were collected by aspiration at 901 points distributed among 11 neighborhoods in São Luís with records of patients with microfilaremia. Pools of engorged or gravid *Culex quinquefasciatus* females were evaluated by *WbCx* duplex PCR with endogenous control for mosquitoes and target for *W*. *bancrofti* for determining the vector infection rate. Among the 10,428 collected mosquitoes, the most abundant species were *C*. *quinquefasciatus* (85%) and *Aedes aegypti* (12%). Significantly larger numbers of mosquitoes were collected from the neighborhoods of Areinha and Coreia (p<0.05). MX performed using PCR validated 705 pools of engorged or gravid females, fifteen of which were positive for *Wuchereria bancrofti* in two neighborhoods.

**Conclusions:**

The high density of engorged *C*. *quinquefasciatus* females per home, inadequate sanitation, and detection of *W*. *bancrofti*-infected mosquitoes in the city of São Luís represent a warning of the possible upsurge of LF, a disease that is still neglected; this underscores the need for the ostensive monitoring of LF in Brazil.

## Author summary

In the diagnosis of lymphatic filariasis (LF) using molecular xenomonitoring, the collection of mosquitoes is a critical step. Catching insects within the residence after the blood meal is ideal because the use of engorged or gravid females increases the odds of finding the parasite ingested during a meal and indicates the occurrence of microfilaremia patients in the region. In São Luís, more than ten thousand adult mosquitoes were removed from the residences in the study period. Considering the confirmation of the pools of *Wuchereria bancrofti*-infected *C*. *quinquefasciatus* females, it is necessary to initiate chemotherapeutic treatment to avoid the recrudescence of LF. Several cities in Brazil have environmental and socioeconomic profiles similar to those found in São Luis, especially those that were considered endemic for LF at some time in the public health history of the country. The collection and diagnostic methods used in the present study can and should be used in other Brazilian states for verifying the interruption of LF transmission.

## Introduction

In 2000, the World Health Organization (WHO) established the Global Programme to Eliminate Lymphatic Filariasis (GPELF), once Lymphatic Filariasis (LF), which is caused by the parasitic nematode *Wuchereria bancrofti*, was established as a Neglected Tropical Disease (NTD) that could be eliminated as a public health problem by 2020 [1]. Of the 81 countries considered endemic for LF at the start of the GPELF, currently, fifty countries and 859 million people remain under the risk of becoming infected with *W*. *bancrofti* and spreading the disease [2]. As the elimination of LF has not yet been achieved globally, the deadlines were revised and a new road map for eliminating this NTD from 2021–2030 was approved by the Seventy-third World Health Assembly in November 2020 [3]. The large-scale annual treatment of humans is one of the cornerstones of the control of LF, as it reduces the load of parasitic microfilariae, because humans are the only known vertebrate reservoir, consequently diminishing the spread of these parasites by mosquitoes [2,4]. For the vigilance of the circulation of LF-causing pathogens, molecular xenomonitoring (MX) has been used to detect the genetic material of filarial worms in samples of mosquitoes through molecular tests, such as polymerase chain reaction (PCR). MX is a noninvasive tool that enables the detection of the presence of parasites in human populations on the basis of their detection in mosquitoes [5]. Since 2002, the WHO has suggested the use of MX for the determination of the interruption of LF transmission [6]. Moreover, improvements in mosquito collection, sampling strategies, and molecular tests have increased the reliability of this approach [7–9].

According to the data from the Brazilian Ministry of Health [10], endemic area of LF in Brazil were reduced to three foci in the 1990s: Metropolitan Region of Recife (MRR) in the state of Pernambuco, the city of Maceió in the state of Alagoas, and Belém, in the state of Pará. Currently, LF is considered to be endemic only to MRR (Recife, Jaboatão dos Guararapes, Olinda and Paulista). However, recent cases of LF were diagnosed among immigrants in the city of Chapecó in the state of Santa Catarina in 2017, which has warned the scientific community of new foci of the circulation of LF in Brazil [11]. Besides the possibility of the introduction of new cases or the expansion of the endemic area, there is a risk of failures in the determination of interruption of transmission, such as those occurring in the city of Maceió (Alagoas). According to Medeiros and his colleagues [12], the error in the determination of Maceió as a focal point with interruption of transmission may have been caused by the lack of planning in the 1951–1970 campaigns and the 1971–2000 programs for the control of filariasis and due to failure in the assessment of epidemiological vigilance. However, after the discovery of autochthonous cases in the region by Dreyer *et al*. [13], the city was reclassified as an endemic area, followed by the establishment of appropriate treatment measures [14].

In 1957, LF was detected in the city of São Luís in the state of Maranhão. Although the elimination of LF was not documented in this city, it has been considered an extinct focal point of LF. In an entomological survey conducted in 1957, São Luís was reported to show the second highest number of female mosquitoes infected with *W*. *bancrofti* (8.2%), after the city of Ponta Grossa (10.8%); the city of Recife was ranked third in this regard (7.3%) [Rachou, 1960 apud 14]. In blood surveys conducted between 1956 and 1965 [Franco and Silva Lima, 1967 apud 12], Maranhão was ranked fifth with regard to the number of people infected with *W*. *bancrofti* (0.24%); the state of Pernambuco was ranked first in this regard (2.66%).

According to the Epidemiological Bulletin of 2016 [15], no other survey was conducted to update the distribution map of LF in Brazil. Available data from few studies underscore the need to reinvestigate the places showing old outbreaks to provide more recent information regarding the status of LF. Once Brazil joined the GPELF in 1997, a dossier containing evidence of the absence of the transmission of LF has to be developed for the validation of its elimination [16]. Therefore, in the present study, due to the lack of evidence for the interruption of *W*. *bancrofti* transmission in Brazil, São Luís (Maranhão) was the first city in the country to be reinvestigated with regard to the status of LF. The mosquito *C*. *quinquefasciatus* is the only vector of *W*. *bancrofti* in Brazil and its presence is closely related with unsanitary conditions, as ditch working as mosquitoes breeding sites [17]. Among the capitals of the Brazilian Northeast, the city of São Luís showed the second lowest percentage of households supplied by the general water distribution network, which in 2010 was 78.53% [18]. In addition, climatic conditions also affect the abundance and distribution pattern of *Culex* mosquitoes [19,20]. Therefore rainfall and socio-economic information were collected to improve our vigilance. In this context, MX was used to generate LF incidence-associated data, provide updated information for the creation of a national dossier regarding the elimination of LF as a public health problem by the year 2030 [3], and deepen our understanding regarding the risk of the resurgence of this neglected disease.

## Methods

### Study area

The entomological survey areas for LF in São Luís were selected by the Secretary of Health and *Laboratório de Endemias do Laboratório Central do Maranhão* (LABEND/LACEN-MA [Endemics Lab of the Central Laboratory of Maranhão]), taking into account the history of LF in the region. The collection of mosquitoes was guided by previous records of patients with microfilaremia in 11 neighborhoods (Table 1). A radius of 100 m was established around each case as the area of assessment. This tracking resulted in the formation of a grid on the local map, on which 50-m quadrants were outlined for the definition of a collection site. Within each quadrant, the household was randomly chosen, the aspiration was performed upon acceptance by the resident, and each household was visited only once. A total of 901 mosquito collection points were established in the city. Distances between collection points were calculated taking into consideration the behavior of *C*. *quinquefasciatus* females in the search for blood meals and preferential breeding sites for egg laying in areas of high population density and precarious sanitary infrastructure, representing endemic areas typical for the spread of LF [9,21]. Thus, 11 neighborhoods of São Luís were identified as entomological survey areas, for which a rainfall report of the collection period was consulted; the hydrological data were obtained from the *Agência Nacional de Águas* (ANA [National Water Agency] (http://www.snirh.gov.br/hidroweb/serieshistoricas). Taking into consideration that breeding sites are influenced by rainfall and that female mosquitoes in the field have an average lifespan of 30 days [22], rainfall during the 30 days prior to the capture of the mosquitoes in each neighborhood was considered. Moreover, socioeconomic data were obtained for these neighborhoods from the *Instituto da Cidade Pesquisa e Planejamento Urbano e Rural* (INCID [Research and Urban and Rural Planning Institute of São Luís] (https://www.agenciasaoluis.com.br/site/arquivodacidade/2236), which is linked to the *Instituto Brasileiro de Geografia e Estatística* (IBGE [Brazilian Institute of Geography and Statistics]) [23].

**Table 1:** Number of mosquitoes collected from different premises in São Luís, MA, Brazil, separated on the basis of species, sex, feeding, and reproductive condition of females.

Neighborhood	*Aedes aegypti*			*Aedes taeniorhynchus*			*Aedes albopictus*			*Culex quinquefasciatus*		
	Male	Female not E/G	Female E/G	Male	Female not E/G	Female E/G	Male	Female not E/G	Female E/G	Male	Female not E/G	Female E/G
ARN	2	4	7	0	0	0	0	0	0	600	200	591
COR	0	0	2	0	0	0	0	0	0	625	290	639
VPA	0	5	2	0	0	0	0	0	1	691	338	753
MCT	73	18	115	0	0	0	2	0	4	858	231	1,014
FAT	15	8	75	2	0	94	0	0	0	80	7	110
LIR	102	8	140	43	0	58	0	0	0	5	1	38
CEN	85	9	201	3	4	65	7	0	0	15	10	31
FAB	43	3	115	0	0	12	0	0	0	87	10	135
LBD	46	1	48	0	0	0	0	0	0	32	6	36
CMB	43	1	35	0	0	0	0	0	0	12	0	15
CDN	4	0	7	0	0	0	0	0	0	519	220	717
Total	**413**	**57**	**747**	**48**	**4**	**229**	**9**	**0**	**5**	**3,524**	**1,313**	**4,079**

Legend: E, engorged (abdomen distended due to blood meal); G, gravid; ARN, Areinha; COR, Coréia; VPA, Vila Passos; MCT, Monte Castelo; FAT, Fátima; LIR, Lira; CEN, Centro; FAB, Fabril; LBD, Liberdade; CMB, Camboa; CDN, Coroadinho. Two regions (Belira and Floresta) are officially a part of other neighborhoods (Centro and Camboa, respectively).

### Mosquito sampling

Mosquito collection is an extremely important step for xenomonitoring. Electric entomological aspirators (Horst Armadilhas - www.horstarmadilhas.com.br) [24] were employed during active searches within the premises chosen for the study, where *C*. *quinquefasciatus* females feed on human blood and search for places to rest. The mosquitoes were collected by field technicians of LABEND/LACEN-MA between August 2016 and October 2017 at the preferred mosquito-resting places for 15 minutes per home during the morning hours (8 to 10 am). The aspirated mosquitoes were taken to LABEND and killed by freezing at -20°C for at least 20 minutes. The mosquito samples were maintained under chilled conditions, examined under a stereoscopic microscope and separated on the basis of the collection date, collection site, species, sex, and physiological conditions, according to the handbook of practical entomology for national lymphatic filariasis elimination programs [5].

All mosquitoes were analyzed; gravid or engorged females of the species *C*. *quinquefasciatus* were pooled and processed for the analysis of LF using MX, given that only blood-fed mosquitoes had the opportunity to be infected by microfilariae [6]. The pooled samples contained up to ten females grouped according to the collection site and were placed in 1.5-mL microtubes with approximately 300 μL of RNA*later* (AMBION). The microtubes were sent to the *Serviço de Referência no Controle de Culicídeos Vetores* (SRCCV [Vector Culicids Control Reference Service]) of the *Instituto Aggeu Magalhães*–Fiocruz–PE. The Annual Biting Rate (ABR) was estimated using the method suggested by the WHO [5]; this was used to calculate the bites per human-hour/days and year. Then, the average numbers of engorged or gravid *Culex* females per premise were divided by the measure of residents per premise per neighborhood (IBGE source).

### Detection of *W*. *bancrofti* DNA via PCR

The sample processing was performed via the following steps: removal of RNA*later*; preparation of macerates of mosquito samples with 500 μL of ultrapure water (without RNase, DNase); extraction of DNA from 200 μL of homogenate, following the protocol described by Ayres et al. [25], and DNA quantification using NanoDrop 2000 spectrophotometer (Thermo Scientific); and *WbCx* duplex PCR with endogenous control for a variety of mosquito genera [26] and target for *W*. *bancrofti*, followed by electrophoresis and documentation according to the protocol described by Albuquerque and colleagues [7]. For avoiding false negatives, the actin target was used as an internal control, and samples for which the amplification did not occur were excluded from the calculation of the vector infection rate (VIR). Although a few *Aedes* species can transmit *W*. *bancrofti* [5], the abundant mosquito *Ae*. *aegypti* in urban environments is refractory to this nematode as demonstrated in an work previously carried out in our laboratory by Magalhães and colleagues [27]. Therefore, only *Culex* mosquitoes were analyzed in this study.

### Data analysis

Homogeneity of variances was determined using the Bartlett test. Differences in the number of adult mosquitoes collected from each neighborhood were tested using Kruskal-Wallis. The median were used to compare differences among treatments applying Fisher’s post hoc test.

To determine the VIR, the data were submitted to the *Poolscreen* statistical model [28], which considers the total number of pools analyzed and the number of pools showing positive results for *W*. *bancrofti*, with a 95% confidence interval (CI). The minimum sample size required to detect positives under particular VIR was calculated using PoolTestR software. The data were processed in the Excel Program (2010 version) and analyzed in the R program version 3.5.0 (2018).

The map of the neighborhoods of São Luís with the entomological survey areas classified according to the number of engorged or pregnant females of *C*. *quinquefasciatus* was constructed using the QGIS 3.10.8 software [29], whose digital cartographic bases were acquired in shapefile format (.shp), in the geodesic system of reference SIRGAS 2000 and in the UTM projection (Universal Transverse Mercator) Zone 23 South. The shapefile files containing the georeferenced graphic information of the city’s neighborhoods and streets were made available by the Municipality of São Luís, through INCID (Institute of the City, Research and Urban and Rural Planning) https://saoluis.ma.gov.br/arquivodacidade/pagina/3472. The shapefile containing information about the city’s hydrography was collected on the website of the National Water Agency (ANA) https://metadados.snirh.gov.br/files/7d054e5a-8cc9-403c-9f1a-085fd933610c/geoft_bho_massa_dagua_v2019.zip.

## Results

### Mosquito density

A total of 10,428 mosquitoes were collected from 901 residences. The two most abundant species were *C*. *quinquefasciatus* (85.5%) and *A*. *aegypti* (11.67%). More than 60% of mosquitoes were Culicidae females and 48.52% were engorged or gravid females (Table 1). Among the 8,916 specimens of *C*. *quinquefasciatus*, 45.75% were engorged or gravid females.

The target sample size calculation was carried out in two stages, in the first one; a total number of mosquitoes were determined in order to obtain the largest possible number of *Culex*. The parameters used were a prevalence of 85%, sampling error of 0.7%. In this way, a total of 9897 mosquitoes was obtained, with a total of culex ranging from (8343 to 8482) for a 95% CI being observed. A prevalence of 60% of females was observed in the *Culex* collected a priori, which is equivalent to 5047 females out of a total of 8412 of *Culex* to be captured. In the second stage, the parameters obtained with the PoolPrev were applied, which was a prevalence of 0.368% and the sampling error of 0.1% obtained a total sample of females N = 3716.

**Fig 1:**
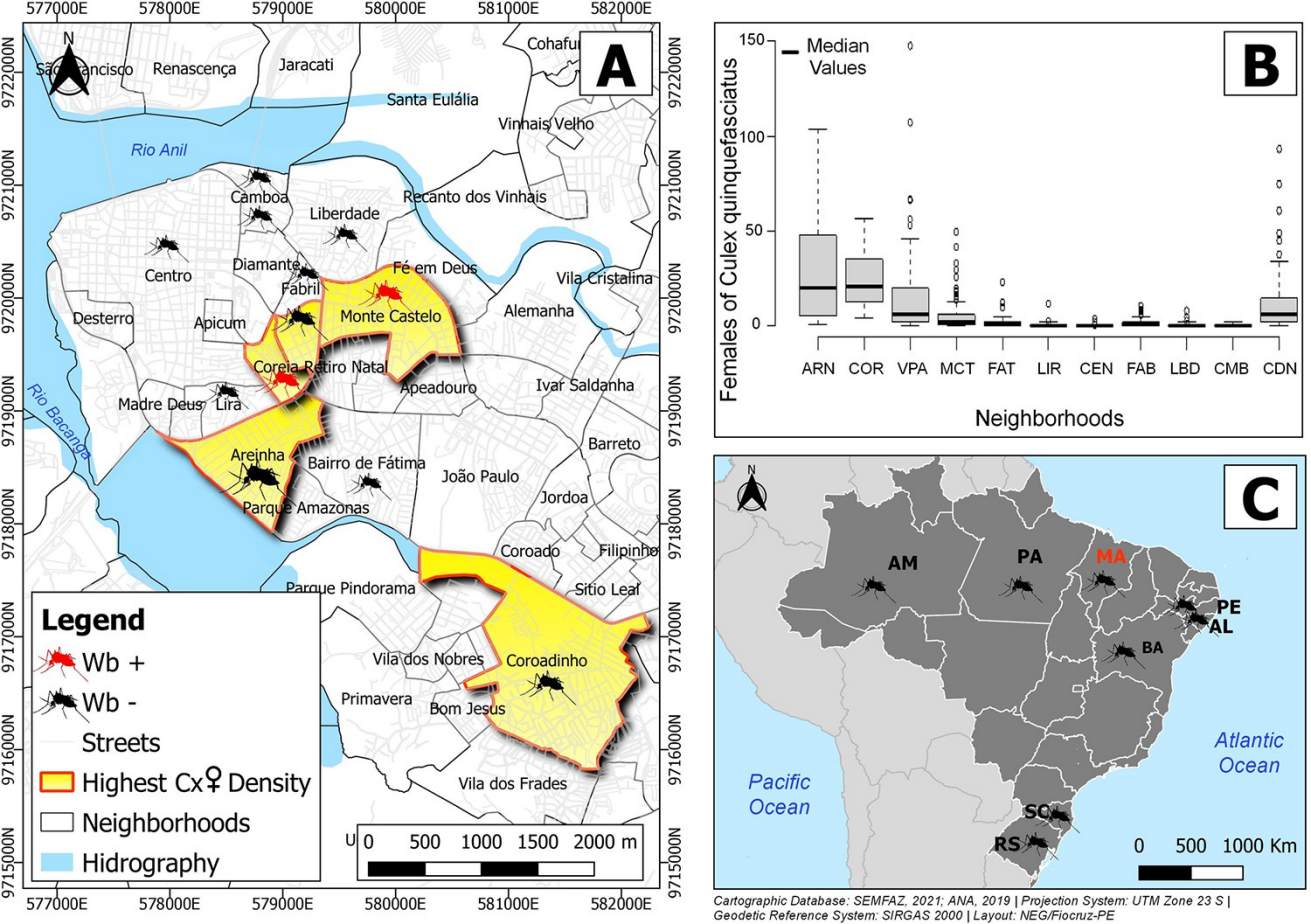
Entomological survey areas ranked with regard to the number of engorged or gravid *C*. *quinquefasciatus* females collected. (A) Map with the neighborhoods of São Luís: ARN—Areinha; COR—Coréia; VPA—Vila Passos; MCT—Monte Castelo; FAT—Fátima; LIR—Lira; CEN—Centro; FAB—Fabril; LBD—Liberdade; CMB—Camboa; CDN—Coroadinho. In red: five neighborhoods with the highest number of engorged or gravid *C*. *quinquefasciatus* females. Red mosquitoes: pools positive for *Wb*; Black mosquitoes: pools negative for *Wb*. (B**)** Means and standard deviations of the numbers of *C*. *quinquefasciatus* females collected per home. (C) LF history represented in Brazil map with states and some cities: AL–Alagoas (Maceió); AM–Amazonas (Manaus); BA–Bahia (Castro Alves); MA–Maranhão (São Luís); PA–Pará (Belém); PE–Pernambuco (Recife); RS–Rio Grande do Sul (Porto Alegre); SC–Santa Catarina (Ponta Grossa, Chapecó). Mosquito design by Freepik from https://www.freepik.com/free-vector/mosquito-silhouette-pack-nine_3138780.htm. Neighborhoods data from INCID (https://saoluis.ma.gov.br/arquivodacidade/pagina/3472) and hydrographic from ANA https://metadados.snirh.gov.br/files/7d054e5a-8cc9-403c-9f1a-085fd933610c/geoft_bho_massa_dagua_v2019.zip.

A significant difference in the total number of *C*. *quinquefasciatus* females was observed among the neighborhoods investigated. A significantly higher number of females was found in the neighborhood of Areinha (20 ± 29.86) than in all the other neighborhoods, with the exception of Coreia (20.5 ± 15.09). Vila Passos (6 ± 27.46) did not differ from that found in the neighborhood of Coroadinho (6± 17.45) which differed significantly from the number of females found in the other neighborhoods (p<0.05) (Table 2) (Fig 1).

**Table 2:** Total *Culex quinquefasciatus* females collected in São Luís, MA, Brazil, between July 2016 and October 2017.

Neighborhood	Premises	Min	Max	Mean	Median	SD
Areinha	27	1	104	29.3	20	29.86
Coreia	38	4	57	24.45	20.5	15.09
Vila passos	61	0	147	17.89	6	27.46
Monte castelo	276	0	50	4.51	2	6.19
Fátima	57	0	23	2.05	1	3.71
Lira	52	0	12	0.75	0	1.77
Centro	130	0	4	0.19	0	0.56
Fabril	86	0	11	1.69	1	2.64
Liberdade	52	0	8	0.81	0.5	1.37
Camboa	48	0	2	0.31	0	0.55
Coroadinho	74	0	93	12.66	6	17.45

### Species of culicids per neighborhood and accumulated rainfall

**Fig 2:**
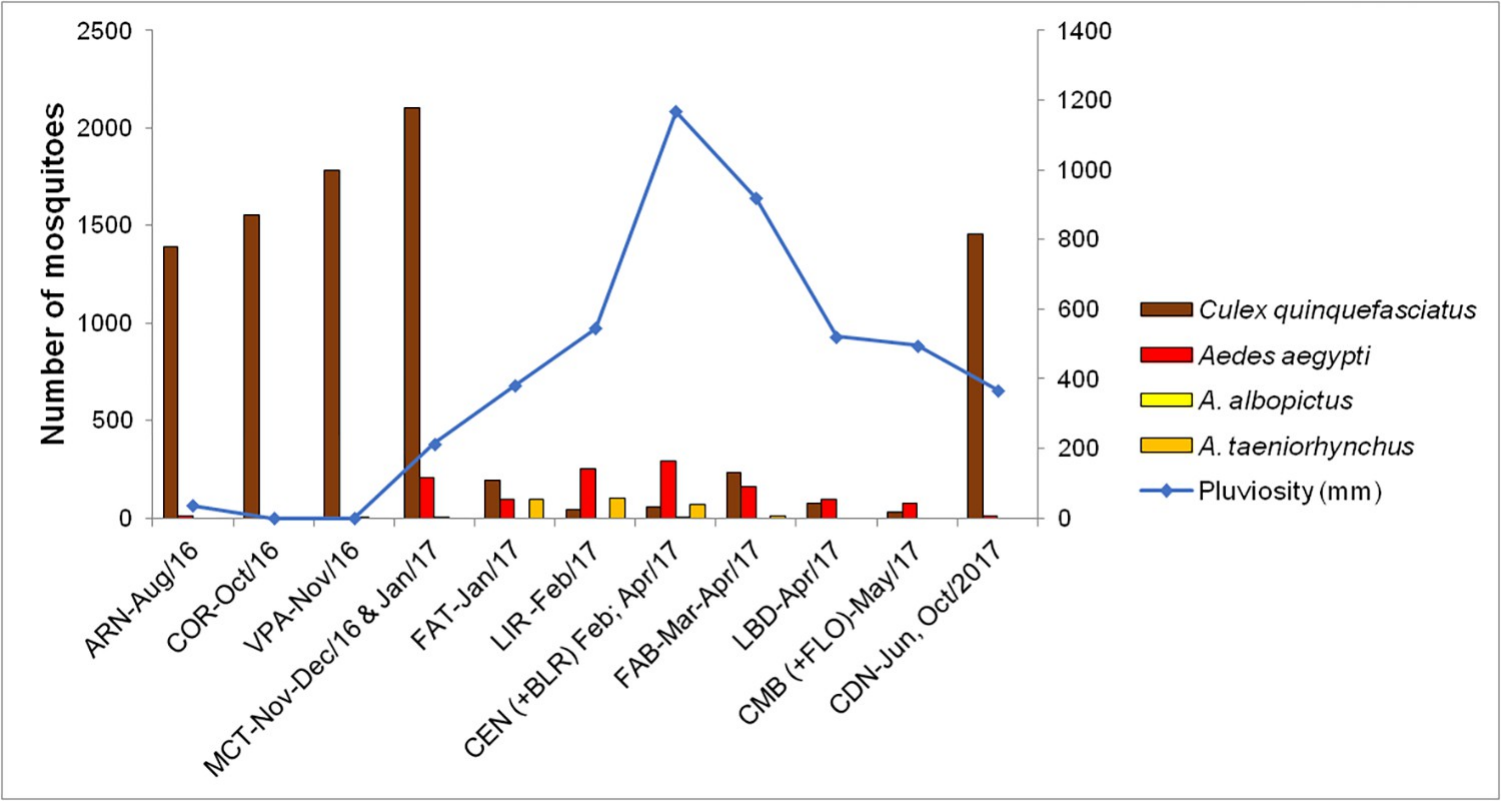
Density of adult mosquitoes collected in the premises sampled in São Luís, MA, Brazil, and accumulated rainfall (30 days prior to the first collection plus days of mosquitoes capture in each neighborhood). ARN, Areinha (33 days); COR, Coréia (33 days); VPA, Vila Passos (46 days); MCT, Monte Castelo (98 days); FAT, Fátima (39 days); LIR, Lira (39 days); CEN, Centro + BLR, Belira (85 days); FAB, Fabril (64 days); LBD, Liberdade (40 days); CMB, Camboa + FLO, Floresta (40 days); CDN, Coroadinho (90 days). Hydrological Information System, *Agência Nacional de Águas* (ANA [National Water Agency]) http://www.snirh.gov.br/hidroweb/serieshistoricas [30].

With regard to rainfall between July 2016 (30 days prior to first collection) and October 2017, the period with the greatest accumulation of rains was January to May 2017, with values higher than 300 mm per month. An increase in the density of *A*. *aegypti* was found in the same period, whereas *C*. *quinquefasciatus* was more frequent in the dry season in the sampled neighborhoods (Fig 2).

### Number of *Culex quinquefasciatus* females collected per premise/neighborhoods and socioeconomic data

Among the neighborhoods selected based on the history of LF, socioeconomic data, obtained as described by the IBGE, was available for ten neighborhoods (Table 3). The neighborhoods that stood out due to the occurrence of premises with a precarious or nonexistent sewage system (ditch, rudimentary sump, or absence of bathrooms) were Camboa (25%), Coroadinho (20%), Liberdade (17%), Fátima (16%), and Areinha (11%). Premises with the largest number of engorged or gravid females of *C*. *quinquefasciatus* per night were found (in decreasing order) in the neighborhoods of Vila Passos (107♀), Areinha (84♀), Coroadinho (70♀), Coreia (50♀) and Monte Castelo (42♀). In all neighborhoods, the proportion of engorged or gravid *C*. *quinquefasciatus* females per premise was higher than 60%, with an overall average of 75%.

**Table 3:** Socioeconomic indicators and *Culex quinquefasciatus* ♀ collected in the entomological survey areas for the investigation of LF status in São Luís, MA, Brazil.

Neighbor-hood	Premi-ses^1a^	Area (Km^2^)	Sampled area (Km^2^)	Premises sampled	% of premises without *Cx* ♀ E-G	*Cx* ♀ E-G/ premise Mean (SD)	*Cx* ♀ E-G/ *Cx* ♀	ABR ^4^ E-G-*Cx ♀*
Areinha	1,664	0,73	0,05	27	3,70%	21.9 ± 24.2	75%	210,327
Coreia	772	0,19	0,01	38	0,00%	16.8 ± 13.7	69%	160,622
Vila Passos	528	0,19	0,11	61	16,40%	12.3 ± 18.4	69%	124,799
Monte Castelo	1,23	0,75	0,57	276	21,70%	3.7 ± 5.1	81%	36,443
Fátima	3,903	0,87	0,02	57	40,40%	1.9 ± 3.5	94%	17,576
Lira	1,206	0,24	0,01	52	61,50%	0.7 ± 1.8	97%	6,814
Centro	ND	2,34	0,06	130	80,80%	0.2 ± 0.5	76%	ND
Fabril	534	0,12	0,02	86	47,70%	1.6 ± 2.4	93%	15,047
Liberdade	4,921	0,94	0,01	52	55,80%	0.7 ± 1.1	86%	6,317
Camboa	1124	0,27	0,04	48	72,90%	0.3 ± 0.6	100%	2,879
Coroadinho	5,933	1,79	0,03	74	13,50%	9.7 ± 13.7	77%	88,877

Legend: 1, Number of premises; 2, Number of residents; 3, Number of premises with inadequate sewage system (ditch, rudimentary sump or no bathroom); *Cx* ♀, *C*. *quinquefasciatus* female; E, engorged (abdomen distended due to blood meal); G, gravid; 4, Annual Biting Rate; a, **Source:** City of São Luís—Research and Urban and Rural Planning Institute—INCID–Socioeconomic data—IBGE/2010 https://www.agenciasaoluis.com.br/site/arquivodacidade/2236.

In São Luís, mosquitoes were collected using an aspirator (collection time, 15 minutes), and it was necessary calculate bites per human-hour/days and year. It was only possible to speculate an indirect ABR (range: 2,879–210,327), the average number of engorged or gravid *Culex* females per premise, divided by the measure of residents per premise per neighborhood (IBGE source).

### Rate of infection of *C*. *quinquefasciatus* by *W*. *bancrofti* in São Luís, MA

Among the 705 validated pools (3925 engorged or gravid females) showing the amplification of the endogenous control in the PCR analysis, fifteen pools were positive for *W*. *bancrofti*. Positive samples were detected in two neighborhoods [Coréia (7 pools) and Monte Castelo (8 pools)] (Fig 1), where the vector infection rates were 1.06% (95% CI: 0.65–2.64%) and 0.37% (95% CI, 0.23–0.89%), respectively. The other neighborhoods had a null vector infection rate (0%) (Table 4).

**Table 4:** Molecular xenomonitoring of the status of LF in São Luís, MA, Brazil.

Neighbor-hood	Year	Period of collection	FemalesE-G	Pools	♀/Pool(1–10)	VIR (%)
Areinha	2016	August	514	65	7.9	0
Coreia	2016	October	582	69	8.4	1.06
Vila Passos	2016	November	753	99	7.6	0
Monte Castelo	2016/17	Nov/Dec/Jan	1022	220	4.6	0.37
Fátima	2017	January	110	36	3.1	0
Lira	2017	February	37	21	1.8	0
Centro (+Belira)	2017	Feb; Abril	122	41	3.0	0
Fabril	2017	Mar/April	34	21	1.6	0
Liberdade	2017	April	23	18	1.3	0
Camboa (+Floresta)	2017	May	15	12	1.3	0
Coroadinho	2017	Jun; Oct	713	103	6.9	0

Legend: E, engorged (abdomen distended due to blood meal); G, gravid; VIR, vector infection rate.

## Discussion

The detection of 15 pools of *C*. *quinquefasciatus* females that were positive for *W*. *bancrofti* in two neighborhoods of São Luis demonstrates the circulation of this pathogen in that area and underscores the need for more precise monitoring of LF in the human population. This finding also supports the hypothesis of the presence of individuals with few or no symptoms that may serve as reservoirs for microfilariae, and as reviewed by Riches [31], it is thought that adults, particularly men, may represent the majority of the reservoir of infection for LF, and studies reported that LF tests show higher prevalence of infection in adults than in children, therefore, the TAS could include adults to the integrated surveillance with xenomonitoring. Furthermore, it is not possible to ascertain whether these findings represent unrecognized foci of filariasis in areas thought to have interrupted transmission or represent a new incursion due to travel or migration. Analyzing the factors that contribute to the reestablishment of LF transmission by migration, this hypothesis cannot be excluded, especially if the migrants are from endemic areas to areas with unsanitary conditions and where mosquito control measures are lacking [32]. A similar situation also was identified in a survey by Pretrick in a region in Satawal Island that was thought to have almost achieved the elimination of LF [33]; in this survey, culicine mosquitoes were found to be positive for *W*. *bancrofti*. The identification and tracking of these hidden foci remains challenging to the control programs for the eradication of filariasis; this confirms the need for long-term post-treatment vigilance to determine the elimination status of LF.

In São Luís, we cannot evaluate the sensitivity of MX screening of the 3933 engorged or gravid females collected in 569 premises, because there were no recent data about human mf prevalence. According to Pryce and Reimer [34], the number of mosquitoes can be determinant for MX, mainly in areas where low mf prevalence in humans is observed, but 100% sensitivity can be observed with sample sizes of 1000 or more. However, when human prevalence is very low (<0.25%), samples of 4000 to 6000 mosquitoes are required to achieve 100% sensitivity. Another data that is not very clear in MX is the size of the area that must be collected related with the number of houses. The WHO [6] recommends the collection of 10 to 50 mosquitoes per house, with 100 to 250 households, while the positive neighborhood Monte Castelo in São Luís has less than 10 mosquitoes per premise in the most of the pools. Therefore, the MX will be strengthened with the work and analysis that will help to build a clear guide that is adaptable and affordable to different field conditions.

Another circumstance that should be considered is that São Luís is a city with many other diseases, such as leprosy, leishmaniasis, arboviruses, schistosomiasis, and tuberculosis [35]; thus, in a region with these many comorbidities, mild filariasis could be masked by others coexisting diseases. A related background has been reported in South Asia, where a case of coinfection was unveiled: a case of asymptomatic filariasis with SARS-CoV-2 was incidentally detected. Mohamed et al [36] have also reported that filariasis coinfection was detected in patients with various conditions, such as malaria and leprosy. Therefore, it is recommended that a broad differential diagnosis and surveys be utilized in areas with several neglected tropical diseases.

In São Luís, the indirect ABR fluctuates between low to very high (range: 2,879–210,327). Considering the factors that influence the efficient transmission of LF, it is estimated that around 15,500 bites by infective mosquitoes per human per year is necessary to cause microfilaremia [37]. Compared with a survey performed in Tanzania [38], the ABR for *C*. *quinquefasciatus* ranged from 1,494 to 6,535 and the ABR for the three vector species, *C*. *quinquefasciatus*, *Anopheles gambiae*, and *An*. *funestus*, ranged from 6,185 to 20,079. São Luís showed a higher risk of transmission of the parasite, and the cumulative exposure over time could increase the chances of being exposed to an infective bite.

In the present study, the calculation of ABR in São Luís may not be accurate once blood-fed and gravid females were counted together, but a close interaction between mosquitoes and people was found. A study performed in Recife in 1995 by Regis *et al* [17] found that 60–120 mosquitoes/room/night were trapped using a CDC miniature light trap. In São Luís, during the 15-min mosquito capture time, five neighborhoods with more *Culex* per premise, Vila Passos (up to 225), Areinha (up to 148), Coroadinho (up to 143), Coreia (up to 128), and Monte Castelo (up to 102), showed a greater density of female mosquitoes than that in Recife at least 20 years ago when the prevalence of LF was higher in the city.

It is noteworthy that 75% of the *C*. *quinquefasciatus* females collected in the entomological survey areas of São Luís were either engorged or gravid. Intense exposure to bites in the same residence in a single night, as was observed in the neighborhood of Vila Passos (107 ♀), which is near the regions from which samples positive for *W*. *bancrofti* were collected [Monte Castelo (42 ♀) and Coreia (50 ♀)], could increase the likelihood of the dissemination and maintenance of LF transmission in the city [17]. Based on statements by the WHO [5], this finding suggests that no methods to avoid human–vector contact have been taken in São Luís, such as screens, mosquito nets, or repellents, which prevent mosquitoes from performing blood meals within the premises, underscoring an eminent risk of the spread of pathogens transmitted by *Culex*.

Therefore, the concerning unsanitary conditions found in São Luís seem to be fundamental for the maintenance of the mosquitogenic sites found in Coréia, the neighborhood with the second highest number of engorged *C*. *quinquefasciatus* females per premise, with seven *W*. *bancrofti*-positive pools being detected. Coréia is also situated beside Areinha, which was the neighborhood with the highest number of engorged females, posing a potential risk of the dissemination of infection in the human population.

In a study conducted in Ghana, Souza *et al* [39] documented the persistence of the transmission of LF 16 years after mass treatment; an estimated microfilaria prevalence of 1.2% and mosquito infection rate of 0.9% were observed, suggesting that individuals with low microfilaremia could be maintaining the transmission of the disease in the region. Similarly, in a longitudinal study conducted in Sri Lanka, Rao and colleagues [40] found persistent LF nine years after the conclusion of mass treatment, and observed that MX was sensitive for detecting *W*. *bancrofti* persistence, when the prevalence of microfilariae dropped from 0.4% to zero and the mosquito infection rate dropped from 0.75% to 0.23%. These studies reported a VIR similar detection to that in São Luís, i.e., 1.06% and 0.37%; thus, it is possible to deduce that the low prevalence of microfilariae in humans is probably occurring in this city. Mild or undetected infections with low levels of microfilariae could increase the transmission; because extremely high microfilaria densities may be fatal to the vector, thus low levels of microfilariae in asymptomatic patients increases the chances of the mosquitoes surviving the infection with the parasite [5,41]. Then, a long term xenosurveillance should be performed to verify *W*. *bancrofti* elimination in Brazil.

As one of the limitations of the study, it is sometimes difficult for technicians to distinguish between mosquito females that have never fed from those that have recently laid eggs but have not yet had a chance to take a new blood meal, so it is risky to leave out a mosquito with larvae of *W*. *bancrofti* at an advanced stage, only if engorged females are analyzed by XM. Therefore, we suggest that all females be processed in the next entomological surveys.

In the present study, the fact that the mosquitoes were searched within the study premises during the morning period and collected using aspiration favored the collection of numerous engorged or gravid *C*. *quinquefasciatus* females (4,079 of the 8,916 *Culex* in 10,428 mosquitoes). This occurred because the blood meal activity of *Culex* mosquitoes is more intense at night and is followed by the search for shelter and rest the subsequent morning for the digestion of the meal and maturation of the eggs [5,42]. These data highlight the high anthropophagy of *C*. *quinquefasciatus* in Brazil and suggest that the active capture of mosquitoes could also be employed as an LF control method, as it promotes the reduction in human–vector contact.

With regard to the type of mosquito-collection tool, the data in the present study are compatible with findings described by Ramesh [9], who performed aspiration in an endemic region of Brazil with precarious urbanization similar to that found in the entomological survey areas of São Luís and showed that this was an efficient method for collecting engorged or gravid *Culex* females. In a study conducted in Egypt, Farid [8] also collected mosquitoes by aspiration, but at night, and found that between 45 and 50% of the mosquitoes collected were engorged or gravid *C*. *pipiens* females. Gravid traps can be very efficient for sampling *Culex* species [5], however, they have not been well accepted by the residents for installation inside the houses in our previous experience. In addition, the large amount of open sewers in São Luís may represent competitors for the traps and reduce the chance of female mosquito collections.

In studies regarding the flight range of *Culex*, Ramesh *et al* [9] reported an average flight range of 35 m in the city of Olinda (state of Pernambuco); further, in most observations made in Texas (USA), Medeiros [21] found that *C*. *quinquefasciatus* females traveled less than 1 km. The flight range of *Culex* females may be strongly linked to the availability of food (preferably, human blood in Brazil), as well as breeding sites rich in organic matter; the influence of this combination of factors is very common in regions investigated for LF, such as Olinda and São Luís [42]. As we found several premises in these communities that had no protection from mosquitoes and precarious or even nonexistent basic sanitation, mosquitoes needed to only travel short distances to feed and lay eggs. Therefore, the use of a 100-m radius around historical cases of LF as an indicator for the collection of vectors in the present study was sufficient to detect the occurrence of microfilariae that infect vectors in an area where the extinction of the focal point had not been documented.

In São Luís, the density of *C*. *quinquefasciatus* was higher during the dry period. One hypothesis for explaining this fact is that breeding sites had a greater concentration of organic matter in this period and were therefore, more productive for the species. In addition, eggs, larvae, and pupae are carried by the flow of rainwater in open-air canals, streams, and ditches. In a study conducted in Recife, Barbosa and Regis [43] found a greater density of the species at the end of the rainy season, highlighting the persistence of breeding sites even after the rains, as observed in canals and open-air sewage ditches found in São Luís. In contrast, the population of *A*. *aegypti* resurged in the rainy season, probably due to the hatching of eggs that were stored in temporary breeding sites formed by the rains [42].

Another study limitation is that it was not possible to analyze the characteristics of sanitary sewage per house, only per neighborhood, in some of them, there is a large channel along the entire street. We only have data from IBGE 2010. In areas of social insecurity, it is difficult and risky for the health agent to ask so many questions, although removing the mosquitoes from inside the house is a relief.

Fighting against a neglected disease like filariasis is challenging, as the presence of *Culex* in households has already become normal, as well as open sewage channels in front of homes, so it is a fight against poverty, and without effective public policies for a proper sanitation is almost impossible. Beyond this integration with community, field teams, health service and research institutions could decrease challenges of NTD roadmap for 2030 [3].

In conclusion, the present study suggests that MX is a sensitive and a suitable tool to detect the circulation of hidden filarial parasites, enabling the detection of individuals serving as reservoirs of microfilariae. Furthermore, xenosurveillance analyzing 4,000 engorged females of *Culex* is easier and less invasive than collecting blood from 4,000 people, who often refuse to participate in epidemiological surveys; moreover, the removal of mosquitoes appears to be a better benefit from the perspective of the residents and should be more sustainable if implemented in routine of the mosquito control.

## Supporting information

S1 Fig Agarose gel electrophoresis showing: Molecular xenomonitoring by *WbCx* PCR with field samples from Coreia—São Luís. M: 1Kb plus Ladder; 1 negative sample; 2–6 positive samples; 7–9 negative samples; 10 positive control from known field sample; 11 negative control from known field sample; 12–13 *Wb*–positive control; N- Negative control.
(PDF)

S2 Fig Agarose gel electrophoresis showing: Molecular xenomonitoring by *WbCx* PCR with field samples from Coreia—São Luís. M: 1Kb plus Ladder; 1–3, 5–7, 9–12: negative samples; 4, 8: not visible bands; 13: positive control from known field sample; 14: *Wb*–positive control; N- Negative control.
(PDF)

S3 Fig Agarose gel electrophoresis showing: Molecular xenomonitoring by *WbCx* PCR with field samples from Camboa—São Luís. M: 1Kb plus Ladder; 1–4, 6–9, 11: negative samples; 5, 10: not visible bands; 12: positive control from known field sample; 13: *Wb*–positive control; N- Negative control.
(PDF)

S4 Fig Agarose gel electrophoresis showing: Molecular xenomonitoring by *WbCx* PCR with field samples from Centro—São Luís. M: 1Kb plus Ladder; 2–3, 5, 7–12: negative samples; 1, 4, 6: not visible bands; 13: positive control from known field sample; 14: *Wb*–positive control; N- Negative control.
(PDF)

S5 Fig Agarose gel electrophoresis showing: Molecular xenomonitoring by *WbCx* PCR with field samples from Fabril—São Luís. M: 1Kb plus Ladder; 1–12: negative samples; 13: positive control from known field sample; 14: *Wb*–positive control; N- Negative control.
(PDF)

S6 Fig Agarose gel electrophoresis showing: Molecular xenomonitoring by *WbCx* PCR with field samples from Coroadinho—São Luís. M: 1Kb plus Ladder; 1–24: negative samples; 25: no sample; 26: *Wb*–positive control; N- Negative control.
(PDF)

S7 Fig Agarose gel electrophoresis showing: Molecular xenomonitoring by *WbCx* PCR with field samples from Fatima—São Luís. M: 1Kb plus Ladder; 1–10: negative samples; 11: positive control from known field sample; 12: *Wb*–positive control; N- Negative control.
(PDF)

S8 Fig Agarose gel electrophoresis showing: Molecular xenomonitoring by *WbCx* PCR with field samples from Vila Passos—São Luís. M: 1Kb plus Ladder; 1–6, 8–10: negative samples; 7: not visible bands; 11: positive control from known field sample; 12: *Wb*–positive control; N- Negative control.
(PDF)

S9 Fig Agarose gel electrophoresis showing: Molecular xenomonitoring by *WbCx* PCR with field samples from Lira—São Luís. M: 1Kb plus Ladder; 1–13: negative samples; 14: positive control from known field sample; 15: *Wb*–positive control; N- Negative control.
(PDF)

S10 Fig Agarose gel electrophoresis showing: Molecular xenomonitoring by *WbCx* PCR with field samples from Monte Castelo—São Luís. M: 1Kb plus Ladder; 1, 3–7, 9–10: negative samples; 2, 8: not visible bands; 11, 13: no sample; 12: *Wb*–positive control; N- Negative control.
(PDF)

S11 Fig Agarose gel electrophoresis showing: Molecular xenomonitoring by *WbCx* PCR with field samples from Monte Castelo—São Luís. M: 1Kb plus Ladder; 1–3: positive samples; 4, 5, 9: negative samples; 7: not visible bands; 6, 8, 10, 12, 14: no sample; 11: positive control from known field sample; 13: Wb–positive control; N- Negative control.
(PDF)

S1 Appendix Number of mosquitoes collected from different premises and neighborhood in São Luís-MA-Brazil. (XLSX)
