# Supplementary material for: Detection of Wuchereria bancrofti in the city of São Luís, state of Maranhão, Brazil: New incursion or persisting problem?
Source: PLoS Negl Trop Dis. 2023 Jan 30;17(1):e0011091. doi: 10.1371/journal.pntd.0011091 (PMC9910792; doi:10.1371/journal.pntd.0011091)
Supplement: S1 Fig — M: 1Kb plus Ladder; 1 negative sample; 2–6 positive samples; 7–9 negative samples; 10 positive control from known field sample; 11 negative control from known field sample; 12–13 Wb–positive control; N- Negative control. (PDF) [file pntd.0011091.s001.pdf]

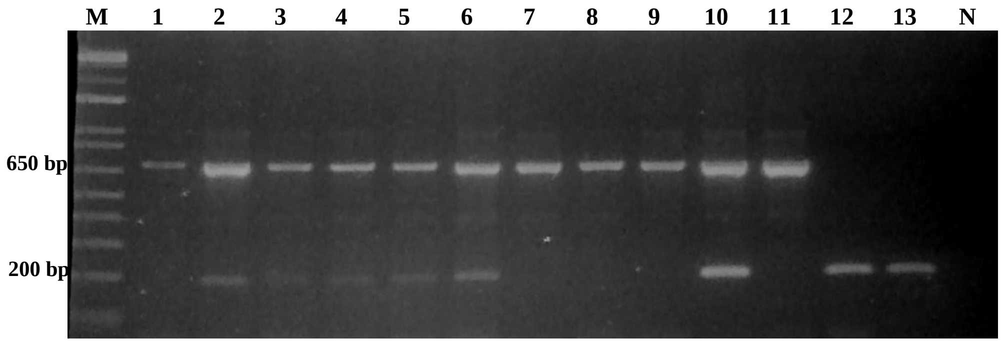

S1 Fig. Agarose gel electrophoresis showing: Molecular xenomonitoring by *WbCx* PCR with field samples from Coreia - São Luís. M: 1Kb plus Ladder; 1: negative sample; 2-6: positive samples; 7-9: negative samples; 10: positive control from known field sample; 11: negative control from known field sample; 12-13: *Wb* – positive control; N- Negative control.
