# Supplementary material for: Detection of Wuchereria bancrofti in the city of São Luís, state of Maranhão, Brazil: New incursion or persisting problem?
Source: PLoS Negl Trop Dis. 2023 Jan 30;17(1):e0011091. doi: 10.1371/journal.pntd.0011091 (PMC9910792; doi:10.1371/journal.pntd.0011091)
Supplement: S6 Fig — M: 1Kb plus Ladder; 1–24: negative samples; 25: no sample; 26: Wb–positive control; N- Negative control. (PDF) [file pntd.0011091.s006.pdf]

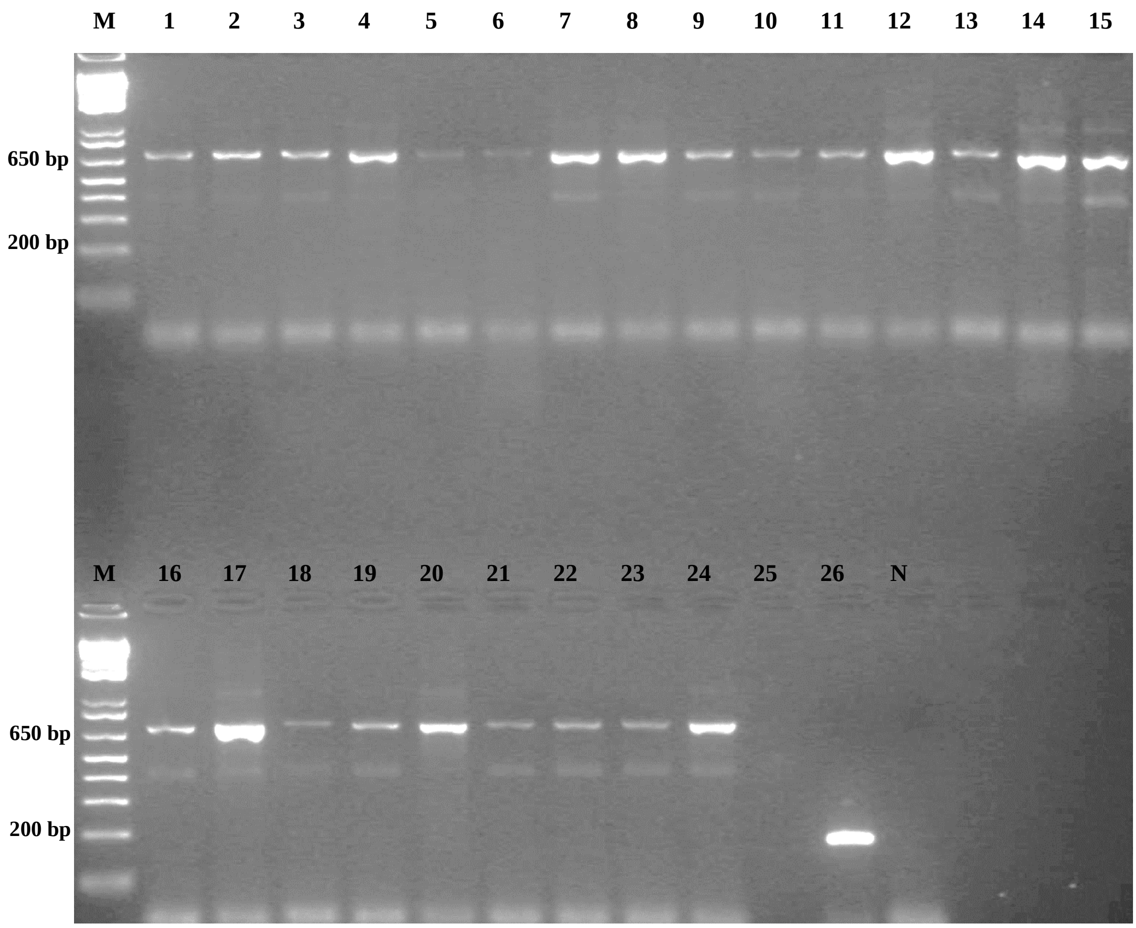

S6 Fig. Agarose gel electrophoresis showing: Molecular xenomonitoring by *WbCx* PCR with field samples from Coroadinho - São Luís. M: 1Kb plus Ladder; 1-24: negative samples; 25: no sample; 26: *Wb* – positive control; N- Negative control.
