# Supplementary material for: Detection of Wuchereria bancrofti in the city of São Luís, state of Maranhão, Brazil: New incursion or persisting problem?
Source: PLoS Negl Trop Dis. 2023 Jan 30;17(1):e0011091. doi: 10.1371/journal.pntd.0011091 (PMC9910792; doi:10.1371/journal.pntd.0011091)
Supplement: S10 Fig — M: 1Kb plus Ladder; 1, 3–7, 9–10: negative samples; 2, 8: not visible bands; 11, 13: no sample; 12: Wb–positive control; N- Negative control. (PDF) [file pntd.0011091.s010.pdf]

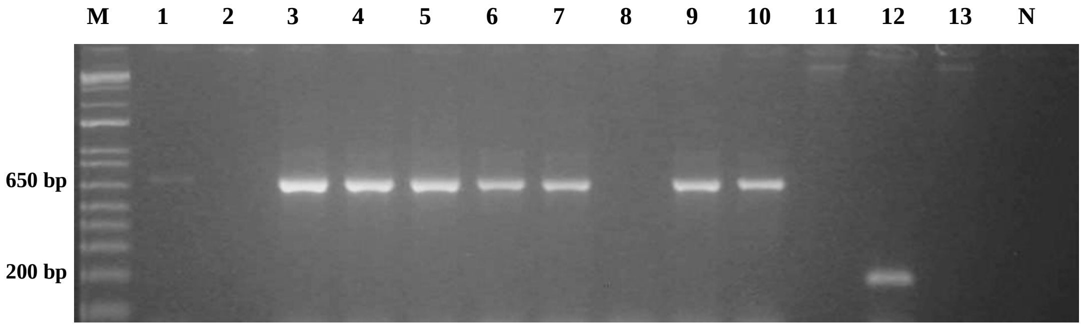

S10 Fig. Agarose gel electrophoresis showing: Molecular xenomonitoring by *WbCx* PCR with field samples from Monte Castelo - São Luís. M: 1Kb plus Ladder; 1, 3-7, 9-10: negative samples; 2, 8: not visible bands; 11, 13: no sample; 12: *Wb* – positive control; N- Negative control.
