# Supplementary material for: Detection of Wuchereria bancrofti in the city of São Luís, state of Maranhão, Brazil: New incursion or persisting problem?
Source: PLoS Negl Trop Dis. 2023 Jan 30;17(1):e0011091. doi: 10.1371/journal.pntd.0011091 (PMC9910792; doi:10.1371/journal.pntd.0011091)
Supplement: S11 Fig — M: 1Kb plus Ladder; 1–3: positive samples; 4, 5, 9: negative samples; 7: not visible bands; 6, 8, 10, 12, 14: no sample; 11: positive control from known field sample; 13: Wb–positive control; N- Negative control. (PDF) [file pntd.0011091.s011.pdf]

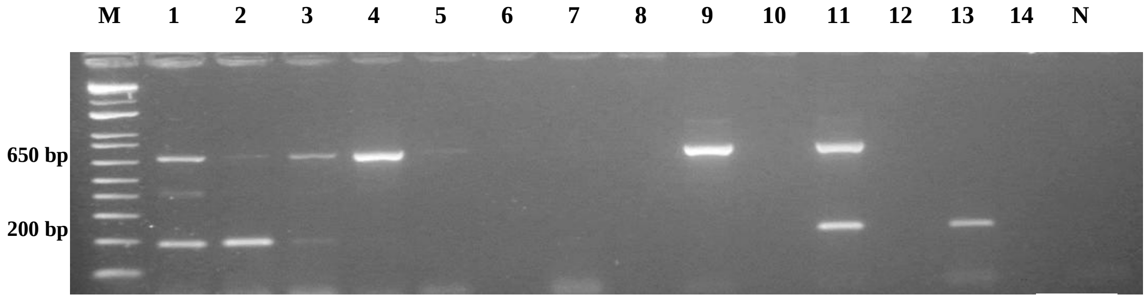

S11 Fig. Agarose gel electrophoresis showing: Molecular xenomonitoring by *WbCx* PCR with field samples from Monte Castelo - São Luís. M: 1Kb plus Ladder; 1-3: positive samples; 4, 5, 9: negative samples; 7: not visible bands; 6, 8, 10, 12, 14: no sample; 11: positive control from known field sample; 13: *Wb* – positive control; N- Negative control.
